# Frailty Index-laboratory and lymphocyte subset patterns in predicting 28-day mortality among elderly sepsis patients: a multicenter observational cohort study

**DOI:** 10.3389/fimmu.2025.1624655

**Published:** 2025-07-16

**Authors:** Dongkai Li, Jiatong Hou, Zhan Shi, Xiao Li, Jiahui Zhang, Guoyu Zhao, Xianli Lei, Yawen Xie, Yuefu Wang, Hao Wang, Zhigang Chang, Na Cui

**Affiliations:** ^1^ Department of Critical Care Medicine, Peking Union Medical College Hospital, Beijing, China; ^2^ Surgical Intensive Care Unit, Beijing Shijitan Hospital, Capital Medical University, Beijing, China; ^3^ Department of Critical Care Medicine, Beijing Hospital, Beijing, China; ^4^ Department of Critical Care Medicine, Beijing Jishuitan Hospital, Beijing, China; ^5^ Department of Critical Care Medicine, Beijing Chao-Yang Hospital, Capital Medical University, Beijing, China

**Keywords:** frailty, sepsis, elderly, natural killer cells, lymphocytes

## Abstract

**Background:**

Frailty is associated with poor outcomes in elderly sepsis patients. This study investigated the relationship between Frailty Index-laboratory (FI-lab) and lymphocyte patterns in predicting 28-day mortality among elderly sepsis patients.

**Methods:**

We conducted a multicenter prospective observational study in four tertiary hospitals in Beijing, China. FI-lab was calculated using 24 laboratory parameters. Peripheral blood lymphocyte subsets were measured at ICU admission. Lymphocyte count trajectories were classified into four phenotypes based on patterns during the first 72 hours. The primary outcome was 28-day mortality.

**Results:**

Among 1,197 patients (mean age 74.6 ± 7.4 years), those with high FI-lab risk showed higher mortality (22.2%) than intermediate (12.0%) and low-risk groups (6.1%). Age-stratified analysis demonstrated consistent FI-lab prognostic value in both 65–79 years (OR 2.18) and ≥80 years (OR 2.47) groups. All lymphocyte subset counts were lower in non-survivors, particularly natural killer cells. In multivariable analysis, high FI-lab risk (OR 2.31), APACHE-II scores (OR 1.08), heart rate (OR 1.01), NK cell count (OR 0.994), and pulmonary infection (OR 1.96) independently predicted 28-day mortality. A combined model incorporating these variables showed superior discriminative ability (AUC=0.788) with excellent internal validation (optimism-corrected AUC=0.775).

**Conclusions:**

FI-lab independently predicts mortality in elderly sepsis patients and correlates with lymphocyte abnormalities. When comprehensive immune assessment is unavailable, lymphocyte trajectory patterns offer a practical approach for risk stratification.

## Introduction

Sepsis is a life-threatening organ dysfunction caused by a dysregulated host response to infection, remains a leading cause of morbidity and mortality in critically ill patients, particularly in the elderly population (1). Frailty is increasingly recognized as a critical factor influencing outcomes in elderly patients with sepsis (2, 3). The Frailty Index-laboratory (FI-lab), which utilizes laboratory parameters to quantify frailty, has gained attention as an objective assessment method in various clinical settings (4, 5). Unlike clinical frailty scales that rely on subjective assessments, FI-lab can be calculated using routine laboratory data, making it suitable for emergency and critical care settings. Recent studies have demonstrated the feasibility and clinical utility of implementing frailty assessment tools in emergency departments and critical care units (9–11).

Sepsis induces profound alterations in immune function, characterized by an initial hyperinflammatory response often followed by immunosuppression (6). Lymphopenia, particularly affecting T cells and natural killer (NK) cells, is a hallmark of sepsis-induced immunosuppression and has been associated with adverse outcomes (7). Recent studies have suggested that dynamic changes in lymphocyte counts over time may better reflect immune status and predict outcomes in sepsis than single measurements (8). Different patterns of lymphocyte count trajectories have been associated with varying mortality risks, potentially offering a practical approach for immune assessment when more detailed lymphocyte subset analysis is unavailable. Contemporary research has highlighted the importance of dynamic immune monitoring approaches in sepsis management (12).”

This study was aimed to: (1) evaluate the prognostic value of FI-lab in elderly patients with sepsis; (2) explore the association between FI-lab scores and peripheral blood lymphocyte subset abnormalities; (3) investigate whether combining FI-lab with lymphocyte analysis improves prediction of 28-day mortality; and (4) develop an integrated prognostic model incorporating frailty and immune parameters for elderly sepsis patients.

## Methods

### Study design and participants

We conducted a multicenter prospective observational cohort study from June 2023 to December 2024 across four tertiary hospitals in Beijing, China: Peking Union Medical College Hospital (ethics approval number: K3148, I-22PJ1104), Beijing Shijitan Hospital (ethics approval number: IIT2023-007-002), Beijing Jishuitan Hospital (ethics approval number: K2023-195-00), and Beijing Hospital (ethics approval number: 2023BJYYEC-150-01). The study was registered at chictr.org.cn (registration number: ChiCTR-ROC-17010750, ChiCTR2300074175).

Eligible patients were aged ≥65 years, consistent with the widely accepted definition of elderly individuals in clinical research and geriatric medicine (2, 9), diagnosed with sepsis according to Sepsis-3 criteria (1), had an expected ICU stay of at least 48 hours, and had no history of immunosuppression. We excluded patients who died within 48 hours of admission, had primary or acquired immunodeficiency, had hematological malignancies, or had insufficient data for FI-lab calculation (>30% missing laboratory values). All patients or their legal representatives provided written informed consent.

### Data collection

Demographic data, comorbidities, vital signs, laboratory parameters, and clinical severity scores were collected upon ICU admission. The Acute Physiology and Chronic Health Evaluation II (APACHE-II) score and Sequential Organ Failure Assessment (SOFA) score were calculated within the first 24 hours of ICU admission. Complete blood counts were performed daily for at least three consecutive days. The primary source of infection was determined based on clinical, radiological, and microbiological findings.

### Frailty Index-Laboratory assessment

Following the methodology described by Howlett et al. (4) and Sapp et al. (5), we calculated the FI-lab using 24 laboratory parameters measured within 24 hours of ICU admission. These parameters included complete blood count (hemoglobin, white blood cell count, platelet count), electrolytes (sodium, potassium, chloride, calcium, phosphate, magnesium), renal function (blood urea nitrogen, creatinine), liver function (alanine aminotransferase, aspartate aminotransferase, albumin, total protein, bilirubin), coagulation profiles (prothrombin time, activated partial thromboplastin time), glucose, and inflammatory markers (C-reactive protein, erythrocyte sedimentation rate). Each parameter was assigned a score of 1 if the value was outside the laboratory’s reference range and 0 if within the normal range, with all parameters weighed equally. The FI-lab score was calculated as the ratio of abnormal values to the total number of parameters (range: 0-1), with higher scores indicating greater frailty. Based on previous literature (4, 5, 13) and to ensure balanced groups for analysis, we classified patients into three risk categories: low risk (FI-lab <0.45), intermediate risk (FI-lab 0.45-0.65), and high risk (FI-lab >0.65).

### Lymphocyte subset analysis and trajectory patterns

Peripheral blood lymphocyte subset analysis was performed within 24 hours of ICU admission using flow cytometry to enumerate total lymphocytes, T lymphocytes (CD3+), T helper cells (CD3+CD4+), cytotoxic T cells (CD3+CD8+), B lymphocytes (CD19+), and natural killer (NK) cells (CD3-CD16+CD56+).

Additionally, based on methodology described by Li et al. (8), we analyzed longitudinal patterns of lymphocyte counts during the first 72 hours of ICU admission. Four distinct trajectory phenotypes were identified based on specific criteria: high-declining (α: initial count >1.5×10^9^/L with negative slope over time), stable-medium (β: maintaining counts between 0.8-1.5×10^9^/L with minimal variation), high-increasing (γ: initial count >1.5×10^9^/L with positive slope), and stable-low (δ: maintaining counts <0.8×10^9^/L with minimal variation). These classifications were based on absolute lymphocyte counts from routine complete blood counts measured at 0, 24, 48, and 72 hours after ICU admission.

### Study outcomes

The primary outcome was all-cause mortality within 28 days of ICU admission, regardless of location (ICU, ward, or post-discharge). Patients were followed for 28 days through hospital records and telephone contact when necessary to ensure complete follow-up.

### Statistical analysis

Continuous variables were presented as means ± standard deviations or medians with interquartile ranges. Categorical variables were presented as frequencies and percentages. Comparisons between groups were performed using Student’s t-test or Mann-Whitney U test for continuous variables and chi-square or Fisher’s exact test for categorical variables.

Sample size calculation was performed based on an expected mortality rate of 15% in elderly sepsis patients, with 80% power to detect an odds ratio of 2.0 for high FI-lab risk, requiring approximately 1,100 patients. Our final sample of 1,197 patients exceeded this requirement. As a secondary analysis, we stratified patients into two age groups (65–79 years and ≥80 years) to examine potential differences in FI-lab performance and lymphocyte patterns across age subgroups.

The association between FI-lab risk categories, lymphocyte subset counts, lymphocyte trajectory patterns, and 28-day mortality was analyzed using univariable and multivariable logistic regression models. Variables with *p<*0.1 in univariable analysis were included in the multivariable model using a forward stepwise selection approach. To address potential overfitting concerns, we performed several validation approaches: (1) Internal validation using bootstrap resampling with 1000 replications to assess optimism and model stability; (2) Calculation of variance inflation factors (VIF) to assess multicollinearity between predictors; (3) Application of the rule of 10 events per variable (EPV), ensuring adequate sample size relative to the number of predictors in our final model. Results were presented as odds ratios (OR) with 95% confidence intervals (CI).Results were presented as odds ratios (OR) with 95% confidence intervals (CI).

Receiver operating characteristic (ROC) curve analysis was performed to evaluate the discriminative ability of different predictive models. Differences between AUC values were compared using DeLong’s test. Kaplan-Meier survival curves were constructed to analyze the relationship between FI-lab risk categories and 28-day survival, with differences assessed using the log-rank test. Internal validation of the final prediction model was performed using bootstrap resampling with 1000 replications to assess optimism and calibration. A p-value <0.05 was considered statistically significant. All statistical analyses were performed using R software version 4.1.2.

## Results

### Patient characteristics

**Figure 1:**
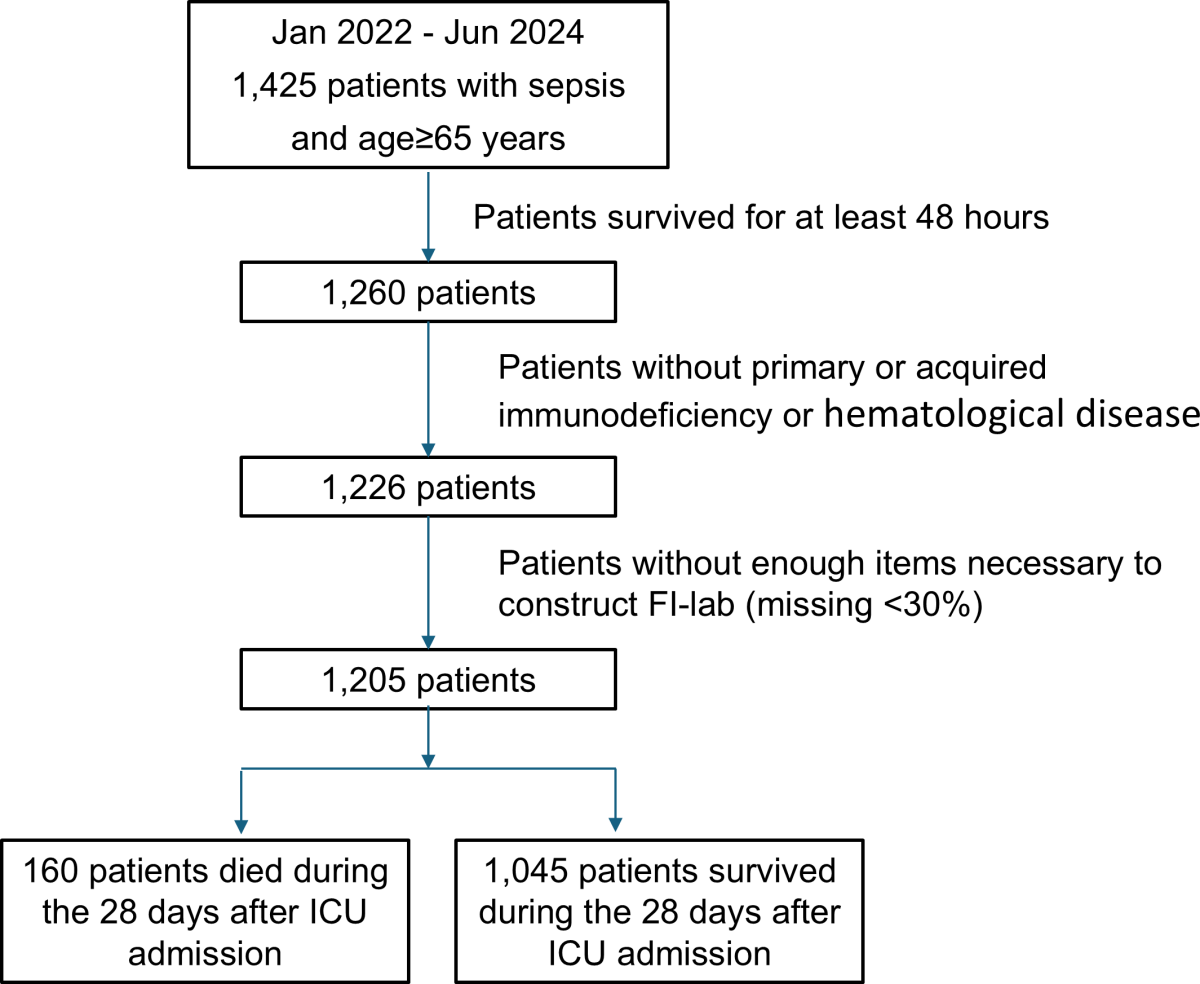
Flowcharts of the study protocol. FI-lab, the Frailty Index based on routine laboratory tests. ICU, Intensive Care Unit.

A total of 1,425 elderly sepsis patients were initially screened, and 1,197 patients meeting the inclusion criteria were enrolled in the final analysis (**Figure 1**). The mean age was 74.6 ± 7.4 years, and 62.7% were male. The primary source of infection was pulmonary in 751 patients (62.7%), followed by abdominal (17.4%), urinary tract (11.3%), soft tissue (5.2%), and others (3.4%). The overall 28-day mortality rate was 13.4% (160/1,197).

**Table 1** compares the characteristics of survivors and non-survivors. Non-survivors had higher APACHE-II scores (26.4 ± 7.6 vs 20.3 ± 6.5, *p<*0.001), higher heart rates (116.5 ± 23.4 vs 104.9 ± 21.4 bpm, *p<*0.001), and were more likely to have pulmonary infections (81% vs 60%, *p=*0.004). No significant differences were observed in age or sex distribution.

**Table 1:** Comparison of clinical characteristics between the 28-day survivors and non-survivors.

Characteristics	Survival (N=1039)	Non-survival (N=158)	*p* Value
Age	74.6 ± 7.6	74.7 ± 7.0	*p=*.908
Sex (Male, %)	654 (62.9%)	96 (60.8%)	*p=*.597
APACHE-II (mean ± SD)	20.3 ± 6.5	26.4 ± 7.6	*p<*.001
SOFA (mean ± SD)	7.2 ± 3.0	8.2 ± 3.8	*p=*.871
HR (bpm, mean ± SD)	104.9 ± 21.4	116.5 ± 23.4	*p<*.001
T (°C, mean ± SD)	36.7 ± 1.0	37.2 ± 1.2	*p=*.133
RR (/min, mean ± SD)	17.5 ± 7.2	20.5 ± 9.5	*p=*.147
OI (mmHg, median ± SD)	242.9 ± 138.7	198.0 ± 143.8	*p=*.172
Lactate (mmol/L, median ± SD)	2.9 ± 2.7	4.0 ± 4.1	*p=*.074
Admission Source			
Elective Surgical	356 (34.3%)	24 (15.2%)	
Medical	417 (40.1%)	108 (68.4%)	*p=*.027
Emergency Surgical	266 (25.6%)	26 (16.5%)	*p=*.593
Pulmonary Infection			
No	416 (40%)	30 (19%)	
Yes	623 (60%)	128 (81%)	*p=*.004
Lymphocyte trajectory phenotype			
δ	325 (31.3%)	69 (43.7%)	
α	65 (6.3%)	15 (9.5%)	*p=*.792
β	582 (56%)	65 (41.1%)	*p<*.001
γ	67 (6.4%)	9 (5.7%)	*p=*.227
BLC (x10^6^/L, mean ± SD)	135.7 ± 142.4	127.2 ± 192.6	*p=*.812
TLC (x10^6^/L, mean ± SD)	544.7 ± 329.9	470.1 ± 361.3	*p=*.068
CD4+TLC (x10^6^/L, mean ± SD)	326.0 ± 206.3	281.1 ± 215.9	*p=*.077
CD8+TLC (x10^6^/L, mean ± SD)	202.2 ± 211.5	154.5 ± 146.5	*p=*.332
NK LC (x10^6^/L, mean ± SD)	104.2 ± 94.2	70.6 ± 82.9	*p=*.050
CD4/CD8 (mean ± SD)	2.4 ± 2.0	2.7 ± 2.2	*p=*.414
FI-lab, mean ± SD	0.57 ± 0.13	0.66 ± 0.12	*p=*.0.23
FI-lab, risk			
Low frailty risk, n (%)	417 (40.1%)	27 (17.1%)	
Intermediate frailty risk, n (%)	314 (30.2%)	43 (27.2%)	*p=*.189
High frailty risk, n (%)	308 (29.6%)	88 (55.7%)	*p=*.001

APACHE-II, Acute Physiology and Chronic Health Evaluation II; HR, heart rate; T, temperature; RR, respiratory rate; OI, oxygenation index; BLC, B-lymphocyte count; TLC, T-lymphocyte count; NK LC, natural killer lymphocyte count; FI-lab, the Frailty Index based on routine laboratory tests.

### FI-lab distribution and association with mortality

The mean FI-lab score was 0.59 ± 0.16. Based on our predefined thresholds guided by previous studies, patients were classified into three approximately equal-sized groups: 444 patients (37.1%) as low risk (FI-lab <0.45), 357 patients (29.8%) as intermediate risk (FI-lab 0.45-0.65), and 396 patients (33.1%) as high risk (FI-lab >0.65). The baseline characteristics of patients stratified by FI-lab risk categories are presented in **Table 2**. Patients in the high-risk group were older, had higher APACHE-II and SOFA scores, higher heart rates, and higher lactate levels compared to the low and intermediate-risk groups (all *p<*0.001). There were no significant differences in sex distribution or source of infection across FI-lab risk categories.

**Table 2:** Comparison of clinical characteristics among the phenotypes of patients in the retrospective cohort.

Characteristics	Low Risk (N=444)	Medium Risk (N=357)	High Risk (N=396)	*p* Value
APACHE-II (mean ± SD)	18.5 ± 6.0	21.1 ± 6.3	24.0 ± 7.3	*p<*.001
SOFA (mean ± SD)	6.3 ± 2.8	7.6 ± 2.7	8.1 ± 3.5	*p<*.001
HR	99.7 ± 19.6	107.6 ± 23.0	113.1 ± 21.6	*p<*.001
T (°C, mean eaSD)	36.6 ± 0.9	36.8 ± 1.0	36.9 ± 1.1	*p<*.001
RR (/min, mean ± SD)	16.8 ± 6.9	18.0 ± 7.4	18.9 ± 8.2	*p<*.001
OI (mmHg, median ± SD)	247.8 ± 126.8	237.0 ± 160.9	224.8 ± 133.3	*p=*.045
Lactate (mmol/L, median ± SD)	2.2 ± 1.8	2.9 ± 2.6	4.0 ± 3.8	*p<*.001
Admission Source				
Elective Surgical	154 (34.7%)	113 (31.7%)	113 (28.5%)	
Medical	181 (40.8%)	164 (45.9%)	180 (45.5%)	*p=*.063
Emergency Surgical	109 (24.5%)	80 (22.4%)	103 (26%)	*p=*.400
Pulmonary Infection				
No	159 (35.8%)	131 (36.7%)	156 (39.4%)	
Yes	285 (64.2%)	226 (63.3%)	240 (60.6%)	*p=*.426
BLC (x10^6^/L, mean ± SD)	138.8 ± 169.3	141.9 ± 136.4	123.3 ± 137.5	*p=*.454
TLC (x10^6^/L, mean ± SD)	572.2 ± 334.6	564.1 ± 369.1	466.7 ± 290.7	*p=*.003
CD4+TLC (x10^6^/L, mean ± SD)	335.2 ± 205.1	329.3 ± 212.7	294.8 ± 205.4	*p=*.053
CD8+TLC (x10^6^/L, mean ± SD)	231.1 ± 201.6	199.3 ± 223.0	153.3 ± 182.4	*p<*.001
NK LC (x10^6^/L, mean ± SD)	118.1 ± 92.2	96.1 ± 97.5	82.6 ± 87.6	*p<*.001
CD4/CD8 (mean ± SD)	2.1 ± 1.7	2.4 ± 2.2	2.7 ± 2.2	*p<*.001
28-Day Outcome				
Non-survival	417 (93.9%)	314 (88%)	308 (77.8%)	
Survival	27 (6.1%)	43 (12%)	88 (22.2%)	*p<*.001
FI-lab, mean ± SD	0.452 .40.004	0.587 .50.001	0.730 .70.005	*p<*.001
Ratio of missing items for FI-lab (%)	16.6 6.0.9	15.1 5.0.9	16.3 6.0.9	*p=*.130

APACHE-II, Acute Physiology and Chronic Health Evaluation II; HR, heart rate; T, temperature; RR, respiratory rate; OI, oxygenation index; BLC, B-lymphocyte count; TLC, T-lymphocyte count; NK LC, natural killer lymphocyte count; FI-lab, the Frailty Index based on routine laboratory tests.

The FI-lab scores were significantly higher in non-survivors compared to survivors (0.66 ± 0.12 vs 0.57 ± 0.13, *p=*0.023). The 28-day mortality rates increased progressively across FI-lab risk categories: 6.1% (27/444) in the low-risk group, 12.0% (43/357) in the intermediate-risk group, and 22.2% (88/396) in the high-risk group (*p<*0.001). Kaplan-Meier survival analysis demonstrated significant differences in 28-day survival among the three FI-lab risk groups (log-rank test, *p<*0.0001, **Supplementary Figure 1**), with separation of curves becoming evident from day 5 onwards. Complete 28-day mortality data were available for all 1,197 patients (100% follow-up rate). The decreasing numbers at risk in the Kaplan-Meier curves reflect events (deaths) and administrative censoring at day 28, not missing data.

### Lymphocyte subset analysis and association with FI-lab

**Figure 2:**
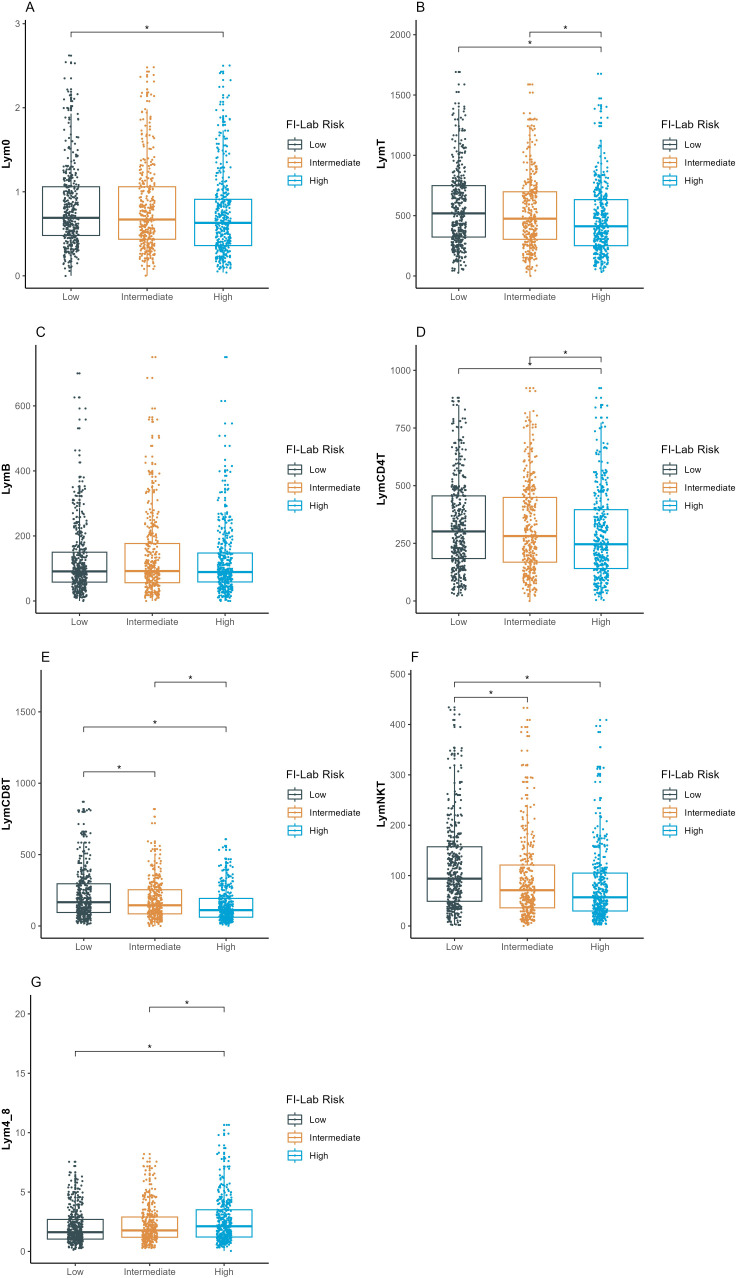
Comparison of lymphocyte subsets count among the three FI-lab risk groups. **(A–G)** represent the different lymphocyte subsets (y-axis). Lym0, total lymphocyte count at intensive care unit admission; LymB, B-lymphocyte count; LymT, T-lymphocyte count; LymCD4T, CD4^+^ T-lymphocyte count; LymCD8T, CD8^+^ T-lymphocyte count; LymNK, natural killer lymphocyte count; Lym4_8, ratio of CD4^+^ to CD8^+^ T lymphocyte counts. The asterisk denotes statistical significance (*P* <.005). FI-lab, the Frailty Index based on routine laboratory tests, MIMIC, Medical Information Mart for Intensive Care; PUMCH, Peking Union Medical College Hospital. ICU, Intensive Care Unit.

Lymphocyte subset counts were compared across FI-lab risk categories (**Figure 2**). All lymphocyte subset counts were progressively lower with increasing FI-lab risk categories (*p<*0.005 for all comparisons). NK cell counts showed the strongest association with FI-lab categories, with mean values of 118.1 ± 92.2 × 10^6^/L in the low-risk group, 96.1 ± 97.5 × 10^6^/L in the intermediate-risk group, and 82.6 ± 87.6 × 10^6/L in the high-risk group (*p<*0.001). The CD4+/CD8+ ratio was significantly higher in the high FI-lab risk group (2.7 ± 2.2) compared to the low-risk group (2.1 ± 1.7, *p<*0.001). Non-survivors had significantly lower lymphocyte subset counts compared to survivors (**Table 1**). Particularly, NK cell counts were markedly lower in non-survivors (70.6 ± 82.9 × 10^6^/L vs 104.2 ± 94.2 × 10^6^/L, *p=*0.050).

### Lymphocyte trajectory patterns and relationship with FI-lab

As our previously published results (8), we verified the four distinct lymphocytes count trajectory patterns: high-declining (α, n=80, 6.7%), stable-medium (β, n=647, 54.1%), high-increasing (γ, n=76, 6.3%), and stable-low (δ, n=394, 32.9%). The consistency of trajectory patterns across all four participating centers (**Supplementary Figure 2**) supports this approach for immune assessment when more detailed lymphocyte subset analysis is unavailable.

The distribution of these trajectory patterns differed significantly among FI-lab risk categories (*p<*0.001, **Supplementary Table 1**). Patients in the high FI-lab risk group were more likely to exhibit the stable-low (δ) trajectory pattern (40.4%) compared to the low-risk group (30.5%). Conversely, patients in the low FI-lab risk group more frequently displayed the stable-medium (β) trajectory pattern (42.3%) compared to the high-risk group (27.2%). Mortality rates also differed significantly among trajectory phenotypes: α (18.8%), β (10.0%), γ (11.8%), and δ (17.5%) (*p=*0.002), with the highest mortality observed in the α (high-declining) phenotype.

### Predictors of 28-day mortality

In univariable logistic regression analysis, APACHE-II scores, heart rate, respiratory rate, lymphocyte trajectory pattern, NK cell count, pulmonary infection, and FI-lab risk category were significantly associated with 28-day mortality (**Table 3**). Multivariable logistic regression analysis identified high FI-lab risk (OR 2.31, 95% CI 1.39-3.84, *p=*0.001), APACHE-II score (OR 1.08 per point increase, 95% CI 1.05-1.11, *p<*0.001), heart rate (OR 1.01 per beat/min increase, 95% CI 1.00-1.02, *p=*0.002), NK cell count (OR 0.994 per 10^6^/L increase, 95% CI 0.990-0.999, *p=*0.046), and pulmonary infection (OR 1.96, 95% CI 1.23-3.14, *p=*0.005) as independent predictors of 28-day mortality. Intermediate FI-lab risk was not significantly associated with increased mortality after adjusting for other factors (OR 1.40, 95% CI 0.82-2.39, *p=*0.218).

**Table 3:** Multivariable logistic regression analysis of predictors of 28-day mortality in the prospective cohort.

Variables	Unadjusted		Adjusted	
	OR (95% CI)	*p* value	OR (95% CI)	*p* value
**APACHE-II**	1.07 (1.04-1.10)	*p<*.001	**1.08 (1.05-1.11)**	***p<*.001**
**HR**	1.01 (1.00-1.02)	*p=*.008	**1.01 (1.00-1.02)**	***p=*.002**
**RR**	1.02 (0.99-1.04)	*p=*.131	1.02 (1.00-1.04)	*p=*.073
**OI**	1.00 (1.00-1.00)	*p=*.212	1.00 (1.00-1.00)	*p=*.170
**Lactate**	1.04 (0.98-1.10)	*p=*.167		
Admission Source				
** Elective Surgical**				
** Medical**	1.95 (1.13-3.36)	*p=*.016	**1.90 (1.13-3.19)**	***p=*.016**
** Emergency Surgical**	1.20 (0.64-2.24)	*p=*.566	1.18 (0.64-2.17)	*p=*.604
Pulmonary Infection				
** No**				
** Yes**	2.00 (1.25-3.21)	*p=*.004	**1.96 (1.23-3.14)**	***p=*.005**
**TLC**	1.00 (1.00-1.00)	*p=*.090		
**CD4+TLC**	1.00 (1.00-1.00)	*p=*.101		
**NK LC**	1.00 (0.99-1.00)	*p=*.067	**1.00 (0.99-1.00)**	***p=*.046**
FI-lab, risk				
** Low frailty risk**				
** Intermediate frailty risk**	1.34 (0.78-2.30)	*p=*.289	**1.40 (0.82-2.39)**	***p=*.218**
** High frailty risk**	2.17 (1.28-3.66)	*p=*.004	**2.31 (1.39-3.84)**	***p=*.001**

*Compared between the patients with pulmonary infection vs. non-pulmonary.

OR, odds ratio; CI, confidence interval; APACHE-II, Acute Physiology and Chronic Health Evaluation II; HR, heart rate; T, temperature; RR, respiratory rate; OI, oxygenation index; TLC, T-lymphocyte count; NK LC, natural killer lymphocyte count; FI-lab, the Frailty Index based on routine laboratory tests.

P values <0.05 were displayed in bold and considered as independent predictors of 28-day mortality.

### Predictive performance of FI-lab and combined models

**Figure 3:**
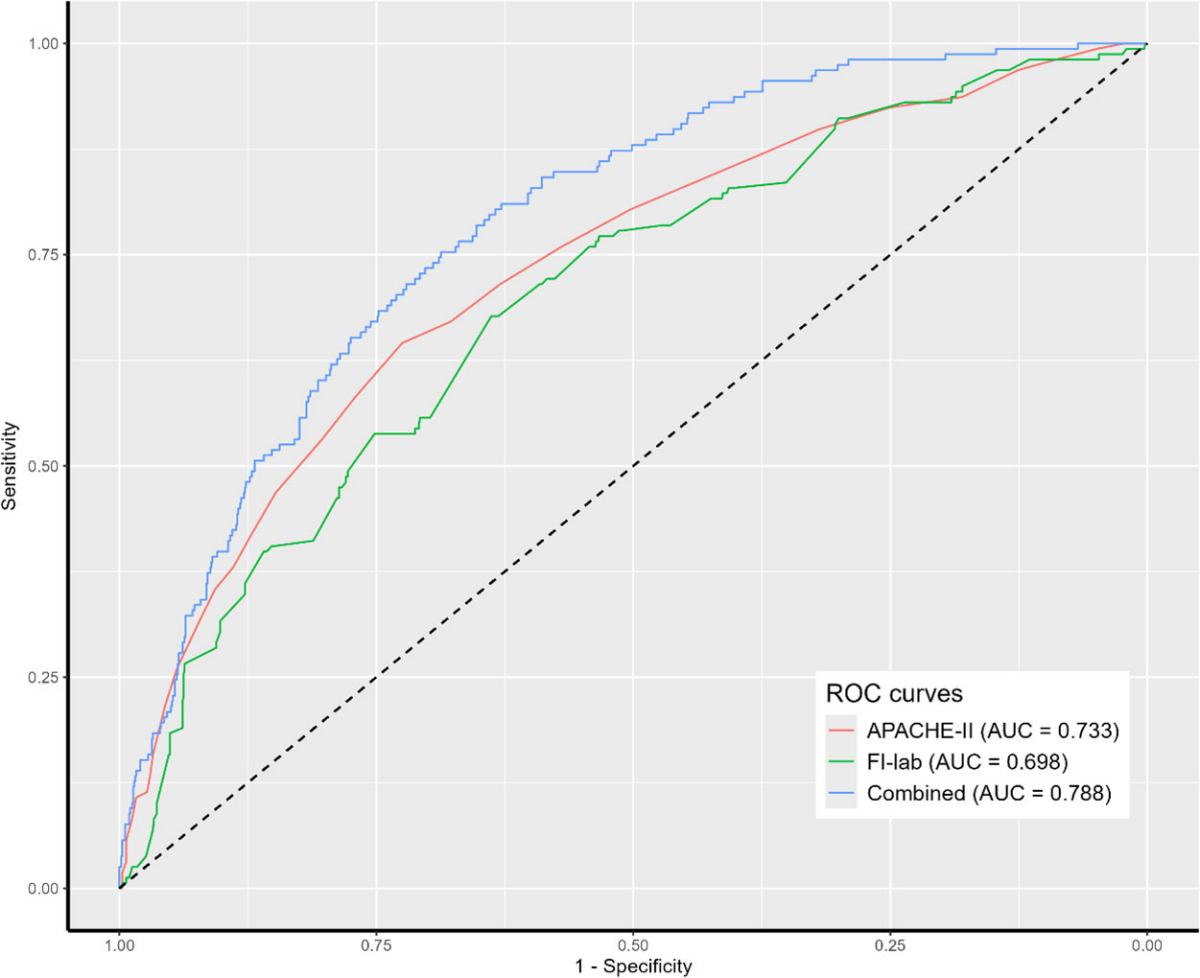
Receiver operating characteristic (ROC) analysis of clinical parameters predicting 28-day mortality. APACHE II, acute physiology and chronic health evaluation II; FI-lab, the Frailty Index based on routine laboratory tests.

ROC curve analysis showed that the APACHE-II scores alone had an AUC of 0.733 (95% CI 0.689-0.777). FI-lab alone demonstrated an AUC of 0.698 (95% CI 0.651-0.745). A combined model incorporating APACHE-II score, FI-lab risk category, NK cell count, heart rate, and source of infection showed improved discriminative ability with an AUC of 0.788 (95% CI 0.748-0.828) (**Figure 3**). DeLong’s test confirmed that the combined model had significantly better discriminative ability compared to either APACHE-II (*p=*0.014) or FI-lab (*p<*0.001) alone.

The bootstrap internal validation demonstrated minimal optimism (optimism-corrected AUC 0.775, 95% CI 0.732-0.817), suggesting good model stability. The shrinkage factor was 0.98, indicating minimal overfitting. With 160 events and 5 predictors in our final model, we achieved an events-per-variable (EPV) ratio of 32, well above the recommended minimum of 10. All variance inflation factors were <2.5, indicating minimal multicollinearity between predictors. Calibration assessment demonstrated reasonable agreement between predicted and observed probabilities of 28-day mortality. A nomogram based on the final multivariable model was developed to provide probability estimates of 28-day mortality (**Supplementary Figure 3**).

### Age-stratified analysis

Among the 1,197 patients, 736 (61.5%) were aged 65–79 years and 461 (38.5%) were ≥80 years. The mean FI-lab scores were higher in the ≥80 years group (0.62 ± 0.15 vs 0.57 ± 0.14, p<0.001). The 28-day mortality rates were 11.1% (82/736) in the 65–79 years group and 16.9% (78/461) in the ≥80 years group (p=0.005). In both age groups, FI-lab remained a significant predictor of mortality after multivariable adjustment (65–79 years: high FI-lab risk OR 2.18, 95% CI 1.15-4.13, p=0.017; ≥80 years: high FI-lab risk OR 2.47, 95% CI 1.28-4.76, p=0.007), demonstrating consistent prognostic value across age subgroups. NK cell counts were similarly predictive in both age groups (65–79 years: OR 0.993, p=0.041; ≥80 years: OR 0.995, p=0.039).

## Discussion

This study demonstrated that FI-lab independently predicts 28-day mortality in elderly patients with sepsis and correlates with lymphocyte subset abnormalities, particularly NK cell depletion. Additionally, lymphocyte trajectory patterns appear useful for immune assessment when detailed lymphocyte subset analysis is unavailable.

### FI-lab in elderly sepsis patients

Our findings support the prognostic utility of FI-lab in elderly sepsis patients, consistent with previous studies in other clinical settings (4, 5, 9). The mean FI-lab score in our cohort (0.59 ± 0.16) was higher than the values reported in hospitalized elderly patients (0.38-0.45) (5), possibly reflecting the increased physiological dysregulation associated with acute sepsis in elderly patients.

The observed increase in mortality across FI-lab risk categories demonstrates the potential value of this tool for risk stratification. FI-lab remained independently associated with mortality after adjusting for established severity scores, which may suggest it captures aspects of vulnerability not fully accounted for in conventional critical care scoring systems. FI-lab offers an objective measure that can be calculated from routinely collected laboratory data, which is a practical advantage for potential implementation in emergencies and critical care settings.

### Frailty and immune dysfunction in sepsis

Our study identified associations between frailty (as measured by FI-lab) and specific immune cell abnormalities in elderly sepsis patients. We observed inverse correlations between FI-lab scores and various lymphocyte subset counts, with the strongest correlation seen for NK cells. These associations may reflect a relationship between physiological frailty and immune competence, though the cross-sectional nature of our analysis prevents determination of causality. These findings are consistent with research suggesting that chronic inflammation associated with aging and frailty may contribute to immune senescence (14). The association between FI-lab and NK cell depletion is also notable. NK cells are known to provide early defense against infections through direct cytotoxicity and cytokine production (15). Recent mechanistic studies have further elucidated the role of NK cell dysfunction in elderly sepsis patients and its therapeutic implications (17). In our multivariable analysis, NK cell counts remained an independent predictor of mortality even after adjusting for FI-lab and APACHE-II scores, suggesting that both frailty and specific immune parameters may have complementary prognostic value.

We also observed an increased CD4+/CD8+ ratio with higher FI-lab scores, suggesting a possibly disproportionate loss of CD8+ T cells in patients with higher frailty scores. This pattern appears to differ from the typical lymphopenia described in sepsis, where both CD4+ and CD8+ T cells are often depleted in similar proportions (7). Current understanding of immunosenescence patterns in sepsis has evolved significantly, with studies providing new insights into age-related immune dysfunction (18). While this observation is interesting, our study design limits our ability to determine whether this represents a frailty-specific pattern of immune dysregulation in sepsis.

### Lymphocyte trajectory patterns for immune assessment

The validation of lymphocyte trajectory patterns described by Li et al. (8) provides a practical approach for immune assessment when detailed analysis is unavailable. The association of different trajectory patterns with FI-lab risk groups and mortality offers insights into the dynamics of immune dysregulation in sepsis. The higher prevalence of the stable-low (δ) pattern in patients with high FI-lab suggests that pre-existing frailty may predispose to persistent immunosuppression during sepsis. Similarly, the association of the high-declining (α) pattern with increased mortality likely reflects rapidly progressing immune dysfunction.

These trajectory patterns were consistent across all four participating centers, suggesting this approach is generalizable. Given that flow cytometry may not be available in all settings, assessment of lymphocyte trajectories using routine complete blood counts offers an accessible method for initial immune assessment.

### Clinical implications

Our findings have several clinical implications. The consistent performance of FI-lab across age subgroups and its integration with routine laboratory data suggest practical implementation potential. Implementing FI-lab calculation in electronic health record systems could help identify high-risk patients who might benefit from closer monitoring. This approach aligns with recent evidence that electronic medical record-based frailty assessment tools can predict adverse outcomes in critically ill older adults (16). Similar findings have been reported in emergency department settings, where frailty assessment tools have shown promise for rapid risk stratification (10, 11). Also, the association between NK cell depletion and mortality, particularly in frailer patients, suggests that NK cell counts may add prognostic value beyond traditional severity scores. The finding that lymphocyte trajectory patterns provide useful prognostic information suggests that even routine complete blood counts, when assessed serially, can aid in risk stratification. The combined model incorporating FI-lab, NK cell counts, and clinical parameters showed improved discriminative ability compared to conventional severity scores alone. The nomogram developed from this model may aid in individualizing risk assessment.

### Limitations

This study has several limitations that should be acknowledged. First, all participating hospitals were tertiary care centers in Beijing, which may limit generalizability to community hospitals or resource-limited settings, though FI-lab’s reliance on routine laboratory parameters supports broader applicability. Second, FI-lab calculations using admission laboratory values may reflect acute illness rather than baseline physiological reserve, and single-point immune measurements cannot fully capture the dynamic evolution of immune dysfunction during sepsis, though our 72-hour lymphocyte trajectory analysis partially addresses this concern. Third, despite excluding patients with known immunosuppression, subclinical immunosenescence and unmeasured comorbidities may have influenced frailty-immune associations. Finally, while internal validation demonstrated good model stability, external validation in diverse healthcare settings is needed before widespread clinical implementation.

### Future directions

Future research should examine changes in FI-lab scores and immune parameters throughout sepsis progression and recovery to better understand their dynamic relationship. Longitudinal studies with pre-admission baseline measurements would help differentiate between chronic frailty and acute illness-induced abnormalities. Implementation studies are needed to determine the feasibility and impact of routine frailty assessment in emergency and critical care workflows. Investigating targeted interventions based on combined frailty and immune profiles may help develop personalized approaches for elderly sepsis patients.

## Conclusion

This study shows that FI-lab independently predicts 28-day mortality in elderly patients with sepsis and correlates with lymphocyte subset abnormalities, particularly NK cell depletion. When detailed immune assessment is unavailable, lymphocyte trajectory patterns offer a practical approach for risk stratification. The combination of frailty and immune assessment may improve prognostic accuracy in elderly sepsis patients.

## Data Availability

The raw data supporting the conclusions of this article will be made available by the authors, without undue reservation.
