# Supplementary material for: Frailty Index-laboratory and lymphocyte subset patterns in predicting 28-day mortality among elderly sepsis patients: a multicenter observational cohort study
Source: Front Immunol. 2025 Jul 16;16:1624655. doi: 10.3389/fimmu.2025.1624655 (PMC12307218; doi:10.3389/fimmu.2025.1624655)
Supplement: Supplementary file 1 [file DataSheet1.pdf]

Supplemental Materials

eTable 1. Comparison of clinical characteristics among the lymphocyte count trajectory phenotypes of patients.

Characteristics	δ (N=394)	α (N=80)	β (N=647)	γ (N=76)	*p* Value
**APACHE-II (mean±SD)**	22.6 ± 7.2	22.2 ± 7.8	20.2 ± 6.4	20.1 ± 7.4	*p*<.001
**SOFA (mean±SD)**	7.6 ± 3.2	6.8 ± 2.9	7.2 ± 3.0	7.3 ± 3.2	*p*=.016
**HR (bpm, mean±SD)**	36.9 ± 1.1	37.0 ± 1.3	36.7 ± 1.0	36.5 ± 0.8	*p*=.024
**T (℃, mean±SD)**	106.2 ± 20.9	110.1 ± 24.3	106.5 ± 22.4	103.6 ± 21.6	*p*=.766
**RR (/min, mean±SD)**	19.2 ± 8.5	17.7 ± 7.7	17.3 ± 7.1	15.9 ± 5.6	*p*<.001
**OI (mmHg, mean±SD)**	222.7 ± 118.4	247.5 ± 209.5	242.6 ± 142.9	252.2 ± 125.7	*p*=.015
**Lactate (mmol/L, mean±SD)**	3.0 ± 2.6	4.2 ± 4.1	2.9 ± 2.9	2.9 ± 3.1	*p*=.905
**Admission Source**					
**Elective Surgical**	93 (23.6%)	28 (35%)	229 (35.4%)	30 (39.5%)	
**Medical**	206 (52.3%)	30 (37.5%)	264 (40.8%)	25 (32.9%)	*p*<.001
**Emergency Surgical**	95 (24.1%)	22 (27.5%)	154 (23.8%)	21 (27.6%)	*p*=.021
**Pulmonary Infection**					
**No**	136 (34.5%)	26 (32.5%)	248 (38.3%)	36 (47.4%)	
**Yes**	258 (65.5%)	54 (67.5%)	399 (61.7%)	40 (52.6%)	*p*=.170
**BLC (x10^6/L, mean±SD)**	78.8 ± 60.3	105.6 ± 90.0	171.6 ± 183.6	139.9 ± 106.8	*p*<.001
**TLC (x10^6/L, mean±SD)**	304.1 ± 169.0	565.0 ± 427.2	664.9 ± 317.4	592.3 ± 388.2	*p*<.001
**CD4+TLC (x10^6/L, mean±SD)**	185.6 ± 125.8	320.6 ± 217.8	395.0 ± 198.7	379.1 ± 265.3	*p*<.001
**CD8+TLC (x10^6/L, mean±SD)**	108.6 ± 85.1	210.7 ± 209.6	246.7 ± 239.1	200.3 ± 170.2	*p*<.001
**NK LC (x10^6/L, mean±SD)**	61.9 ± 58.4	93.0 ± 94.8	122.5 ± 104.1	109.4 ± 80.2	*p*<.001
**CD4/CD8 (mean±SD)**	2.3 ± 2.0	2.5 ± 2.0	2.5 ± 2.0	2.6 ± 2.2	*p*=.306
**28-Day Outcome**					
**Non-survival**	325 (82.5%)	65 (81.2%)	582 (90%)	67 (88.2%)	
**Survival**	69 (17.5%)	15 (18.8%)	65 (10%)	9 (11.8%)	*p*=.002
**FI-lab Risk**					
**Low**	120 (30.5%)	21 (26.2%)	274 (42.3%)	29 (38.2%)	
**Medium**	115 (29.2%)	28 (35%)	197 (30.4%)	17 (22.4%)	*p*=.110
**High**	159 (40.4%)	31 (38.8%)	176 (27.2%)	30 (39.5%)	*p*<.001

Abbreviations: APACHE-II, Acute Physiology and Chronic Health Evaluation II; HR, heart rate; T, temperature; RR, respiratory rate; OI, oxygenation index; BLC, B-lymphocyte count; TLC, T-lymphocyte count; NK LC, natural killer lymphocyte count; FI-lab, the Frailty Index based on routine laboratory tests.

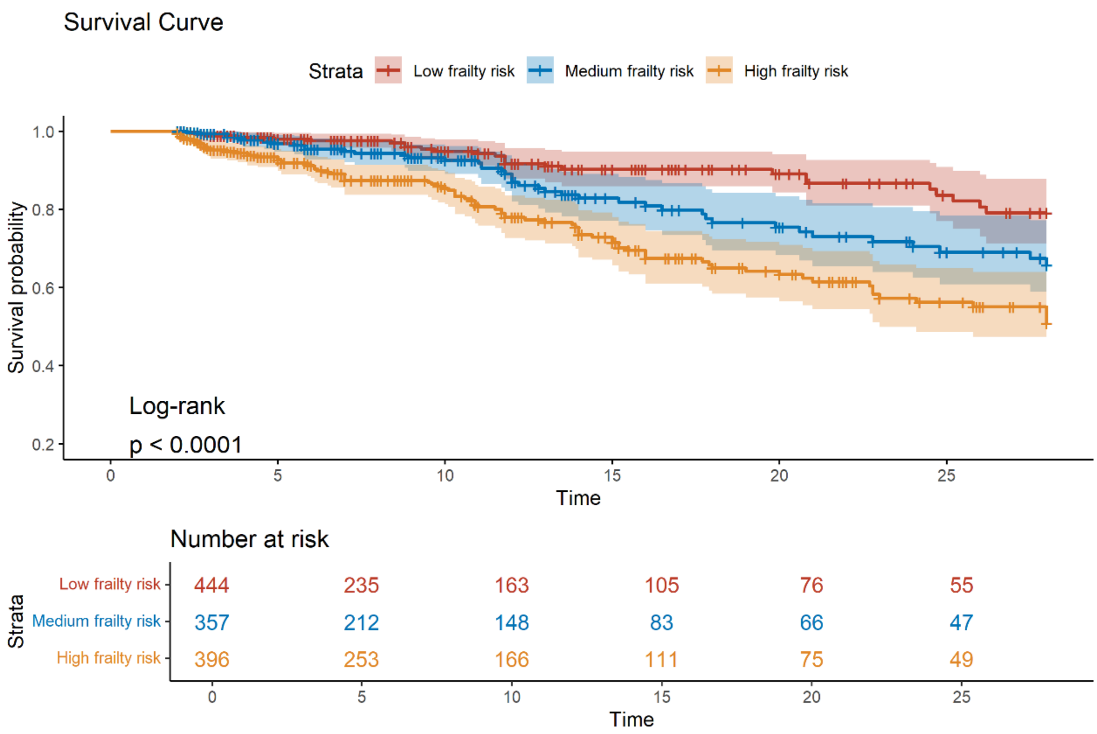

eFigure 1. Survival curves of 28-day mortality by FI-lab risk. The *p* value (log-rank test) for the comparison among the survival curves of the three FI-lab risk groups is shown. Abbreviations: FI-lab, the Frailty Index based on routine laboratory tests.

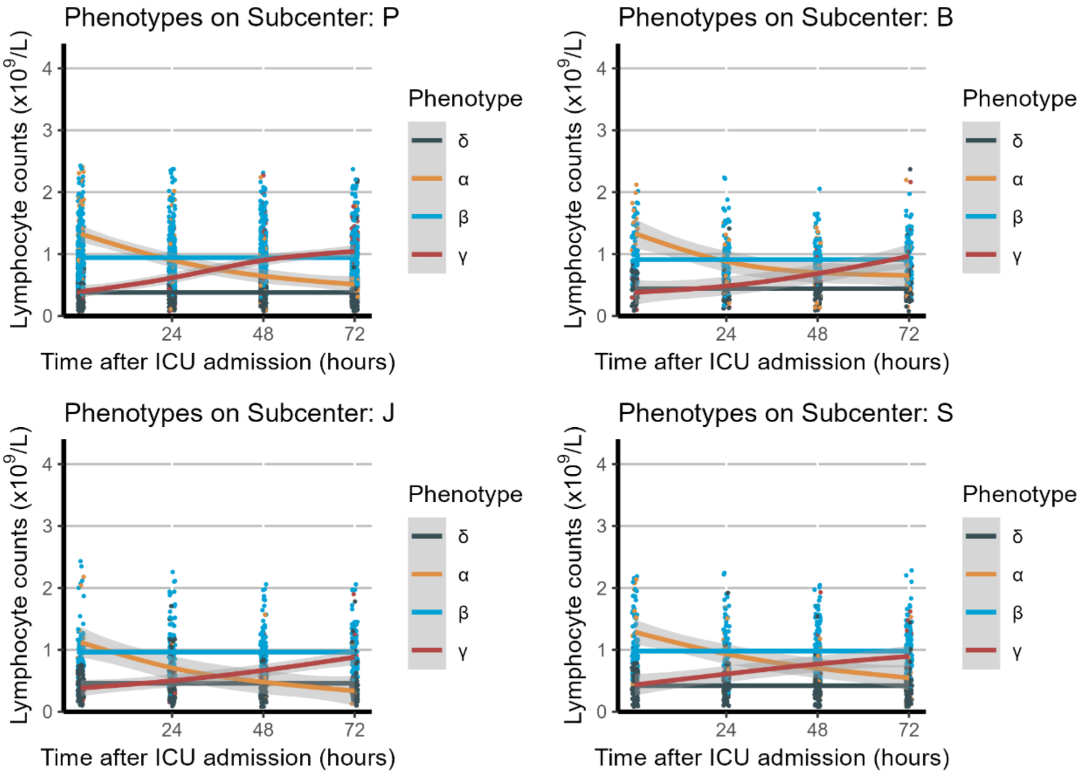

eFigure 2. Demonstration of longitudinal lymphocytes count trajectory phenotypes among the four subcenters of the study. The x-axis represents the timing of lymphocyte measurement (0, 24, 48, and 72 hours after ICU admission), and the y-axis represents the lymphocyte counts (x10^9/L). The shaded area represents the confidence interval around the smooth curve, with a confidence level of 0.95. Abbreviations for the four subcenters: P for Peking Union Medical College Hospital, B for Beijing Hospital, J for Beijing Jishuitan Hospital and S for Beijing Shijitan Hostpial. ICU, Intensive Care Unit.

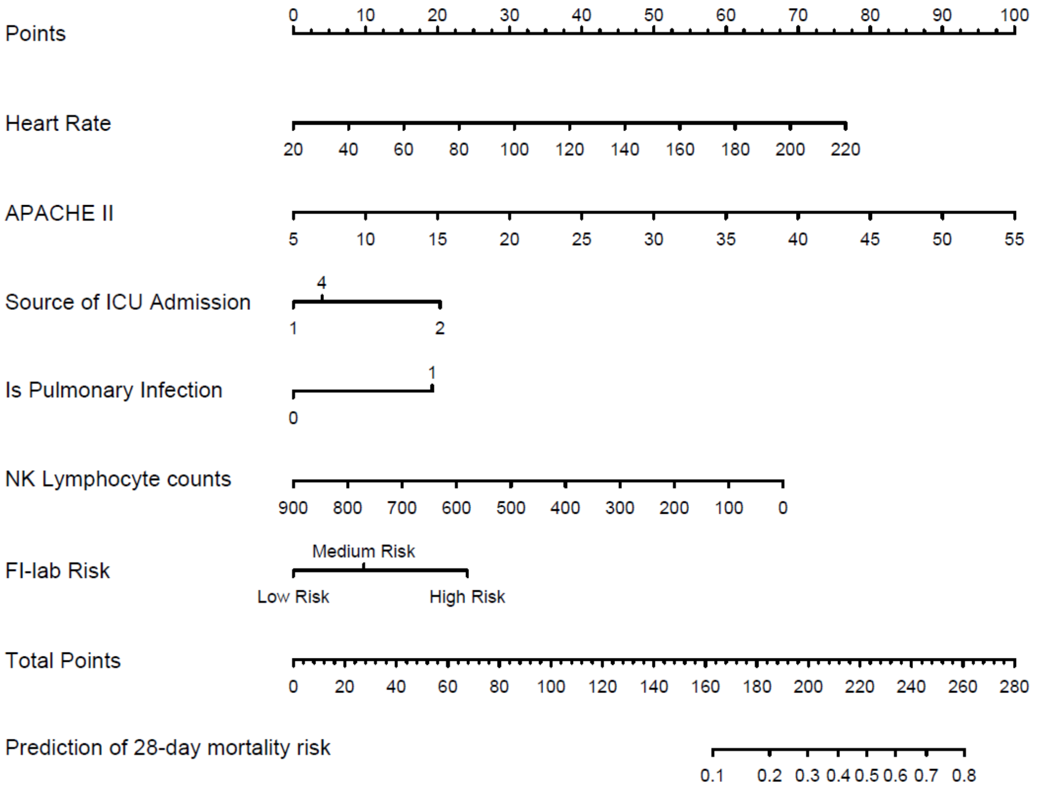

eFigure 3. Characteristics included in the nomogram to predict 28-day mortality in elder patients with sepsis. Abbreviations**:** APACHE-II, Acute Physiology and Chronic Health Evaluation II; HR, heart rate; NK, natural killer cell.
